# Blood Pressure Variability in Acute Stroke: A Narrative Review

**DOI:** 10.3390/jcm13071981

**Published:** 2024-03-29

**Authors:** Christina Zompola, Lina Palaiodimou, Konstantinos Voumvourakis, Leonidas Stefanis, Aristeidis H. Katsanos, Else C. Sandset, Estathios Boviatsis, Georgios Tsivgoulis

**Affiliations:** 1 Second Department of Neurology, “Attikon” University Hospital, School of Medicine, National and Kapodistrian University of Athens, 12462 Athens, Greece; 2 First Department of Neurology, “Aeginition” University Hospital, School of Medicine, National and Kapodistrian University of Athens, 11528 Athens, Greece; 3 Division of Neurology, McMaster University/Population Health Research Institute, Hamilton, ON L8L2X2, Canada; 4 Stroke Unit, Department of Neurology, Oslo University Hospital, N-0424 Oslo, Norway; 5 Second Department of Neurosurgery, “Attikon” University Hospital, School of Medicine, National and Kapodistrian University of Athens, 12462 Athens, Greece

**Keywords:** acute stroke, blood pressure variability

## Abstract

The management of blood pressure variability (BPV) in acute stroke presents a complex challenge with profound implications for patient outcomes. This narrative review examines the role of BPV across various stages of acute stroke care, highlighting its impact on treatment strategies and prognostic considerations. In the prehospital setting, while guidelines lack specific recommendations for BP management, emerging evidence suggests a potential link between BPV and outcomes. Among ischaemic stroke patients who are ineligible for reperfusion therapies, BPV independently influences functional outcomes, emphasising the need for individualised approaches to BP control. During intravenous thrombolysis and endovascular therapy, the intricate interplay between BP levels, recanalisation status, and BPV is evident. Striking a balance between aggressive BP lowering and avoiding hypoperfusion-related complications is essential. Intracerebral haemorrhage management is further complicated by BPV, which emerges as a predictor of mortality and disability, necessitating nuanced BP management strategies. Finally, among patients with acute subarachnoid haemorrhage, increased BPV may be correlated with a rebleeding risk and worse outcomes, emphasizing the need for BPV monitoring in this population. Integration of BPV assessment into clinical practice and research protocols is crucial for refining treatment strategies that are tailored to individual patient needs. Future studies should explore novel interventions targeting BPV modulation to optimise stroke care outcomes.

## 1. Introduction

Hypertension is a significant risk factor for stroke with a 10% increase in its prevalence being correlated with an almost three-fold higher stroke incidence at the population level [1]. However, the elevated blood pressure (BP) levels that are commonly found in the acute setting of stroke can only partially be attributed to a previous history of arterial hypertension [2]. Acute hypertensive response is an expected complication after acute stroke with the capacity to affect not only hypertensive but also previously normotensive patients through different pathophysiological mechanisms [3]. Although in most cases BP tends to normalise within the first hours after stroke, elevated BP levels in the acute phase are independently associated with adverse short- and long-term clinical outcomes. [4]. In addition to elevated BP levels, post-stroke hypotension was also associated with adverse functional outcomes in patients with acute stroke [5]. Thus, managing blood pressure in the context of acute stroke can be challenging, requiring a balanced approach that aims to maintain adequate cerebral perfusion while minimising the risk of complications, depending on individualised circumstances. Despite the currently available guidelines, which provide some general recommendations [6,7], the optimal management of BP in acute stroke has not yet been thoroughly established, and several factors may alter the strategy for BP reduction. Stroke subtype, treatment with acute reperfusion therapies in the case of acute ischaemic stroke, baseline BP levels and comorbidities may all modify the optimal target BP range during the acute phase of stroke. In addition, there is no clear consensus on the optimal antihypertensive agent to be used in each case.

An additional factor that should be considered during BP management is BP variability (BPV) [8], which is defined as the degree of fluctuation in BP values over time that can be expressed through several indices (Table 1) [9]. BPV may act as a third independent component of BP dysregulation in the acute phase of stroke along with hypertension and hypotension. In fact, BPV seems to affect stroke pathophysiology and outcomes via cerebral autoregulation. In the acute phase of stroke, cerebral autoregulation is often impaired and, thus, BP fluctuations have a more direct impact on cerebral perfusion [10]. Fluctuations in BP may result in inadequate perfusion to areas already compromised by the stroke (potentially exacerbating ischaemic injury) and, at the same time, disruption of the blood–brain barrier (potentially promoting haemorrhage) [11,12]. Thus, increased BPV may be a target for treatment, aiming at stable cerebral perfusion and improved stroke outcomes [13].

In this narrative review, the effect of BPV on stroke outcomes is discussed, and the available evidence and the current status of clinical research on BPV are presented in the context of different acute stroke settings: (i) prehospital management; (ii) management of patients with acute ischaemic stroke that are ineligible for reperfusion therapies; (iii) patients with acute ischaemic stroke that are receiving intravenous thrombolysis; (iv) patients with large vessel occlusions that are treated with endovascular therapy; (v) patients with primary intracerebral haemorrhage; and (vi) patients with subarachnoid haemorrhage.

## 2. Methods

For this narrative review, a systematic search on BPV and acute stroke was conducted by two independent researchers (CZ, LP), searching MEDLINE, Scopus and Embase, Cochrane Library, EudraCT (European Union Drug Regulating Authorities Clinical Trials Database) and ClinicalTrials.gov databases. An additional manual search of conference abstracts and reference lists of published articles was also performed to ensure the comprehensiveness of bibliography. The last electronic search was performed on 1 March 2024.

Completed and ongoing randomised controlled clinical trials and prospective or retrospective observational cohort studies that provided clinical data regarding BPV and acute stroke were evaluated, and relevant information was abstracted. Additionally, relevant editorials, commentaries and reviews that provided an overview of BPV effect in acute stroke were also assessed to ensure the comprehensiveness of bibliography. Case series and case reports were excluded for inclusion in this narrative review. Finally, due to the lack of basic scientific data on BPV and stroke [14], preclinical and animal studies were not available for inclusion.

## 3. Blood Pressure Variability in the Prehospital Setting of Acute Stroke

The current American Heart Association/American Stroke Association guidelines do not provide specific recommendations for acute BP lowering in the prehospital setting [6]. The European Academy of Neurology and the European Stroke Association specifically addressed prehospital stroke management and provided a consensus statement that did not recommend prehospital management of high BP in patients suspected of acute stroke [7,15]. However, the quality of the available evidence was very poor, and this recommendation was considered rather weak [7,15].

Several studies were conducted regarding BP management in the prehospital setting of acute stroke. The Rapid Intervention with Glyceryl Trinitrate in Hypertensive Stroke (RIGHT) trial [16], followed by the RIGHT-2 trial [17], evaluated the safety and efficacy of BP lowering by administering transdermal glyceryl trinitrate during prehospital management in patients with suspected stroke; however, results were neutral when transdermal glyceryl trinitrate was compared to placebo for both functional outcomes and mortality at 3 months. Transdermal glyceryl trinitrate was further tested in the prehospital setting during the Multicentre Randomised trial of Acute Stroke treatment in an Ambulance with a nitroglycerin Patch (MR ASAP), which was prematurely terminated due to safety concerns in the subgroup of patients with intracerebral haemorrhage while resulting in no differences regarding efficacy between the two groups in the total population [18]. Another trial, the Paramedic-Initiated Lisinopril for Acute Stroke Treatment (PIL-FAST) trial [19], tested the use of sublingual lisinopril in the same setting but again showed no effect on the short-term prognosis of the patients. The Field Administration of Stroke Therapy-Magnesium trial (FAST-MAG) was a phase 3 randomised controlled clinical trial investigating the prehospital initiation of magnesium for patients with stroke presenting within 2 h from last known well [20]. After enrolling 1700 patients, this study showed that, although prehospital initiation of magnesium sulfate therapy was feasible and safe, it was not associated with functional outcomes and mortality at 90 days.

Regarding BPV and its association with stroke outcomes, only limited data exist for the prehospital setting. It was previously demonstrated that BPV in the prehospital setting does not differ between stroke patients and stroke mimics, based on a retrospective analysis of the electronic records of 960 patients that were transported by the Emergency Medical Services [21]. However, according to the Standard Approach and Ongoing Assessment Medical Control Protocol used in this study, only two sets of BP readings were measured by the personnel every 15 to 30 min, significantly restricting the reliability of the BPV calculation.

Among patients with ischaemic stroke requiring endovascular thrombectomy, a small study, including 134 patients in the prehospital setting and during transportation from a primary stroke center to a mechanical thrombectomy-capable center, examined the association of maximum systolic BP and BPV with stroke outcomes and acute kidney injury [22]. According to the findings of this study, BPV was not associated with functional outcomes post-stroke. On the other hand, maximum systolic BP levels were related to a lower likelihood of mRS (modified Rankin Scale) score ≤ 2 at 3 months. However, this study is limited, not only due to the restricted sample size but also due to the fact that BPV was calculated based on five consecutive BP measurements that were undertaken during the 30 min period of transportation. It is questionable whether BPV within such a restricted time window could have significant associations with long-term outcomes post-stroke.

Finally, in a post-hoc analysis of the FAST-MAG trial, 386 patients with a final diagnosis of intracerebral haemorrhage with available multiple BP measurements in the prehospital and early emergency department phase of care were further evaluated [23]. BPV measured during the hyperacute period was expressed by standard deviation, coefficient of variation and successive variation, all of which were associated with poor outcomes at 3 months, independently of mean or maximum systolic BP [23]. Based on these findings, it was postulated that stabilisation of BPV during the hyperacute period may be a promising therapeutic target. Interestingly, in a more recent, secondary analysis of the FAST-MAG trial, it was shown that BPV was not associated with haematoma expansion among patients with intracerebral haemorrhage [24]. Therefore, the negative association of elevated BPV and stroke outcomes seems to be driven by factors other than haematoma expansion, which could include secondary ischaemia or perihematomal edema.

Further research is being conducted regarding hyperacute BP lowering in the prehospital setting. Administration of urapidil is being tested against as the standard of care in the Intensive Ambulance-delivered Blood Pressure Reduction in Hyper-Acute Stroke (INTERACT4) trial [25]. Although BPV is not being investigated according to the protocol of this trial, several consecutive measurements of systolic BP levels will be conducted, allowing for the calculation of BPV indices. The investigators are urged to further explore the potential interaction of BPV at least as a post-hoc analysis, since BPV data in the prehospital setting are scarce.

## 4. Blood Pressure Variability among Patients with Acute Ischaemic Stroke That Are Ineligible for Reperfusion Treatment

In the absence of specific reperfusion treatments, management and normalisation of the vital signs, including BP levels, is of paramount importance among patients with ischaemic stroke that are not eligible for either intravenous thrombolysis or endovascular treatment. Several large, randomised controlled clinical trials assessing BP management in the acute phase of stroke have included patients that did not receive any acute reperfusion treatments. Among them, the Scandinavian Candesartan Acute Stroke (SCAST) trial evaluating intravenous administration of candesartan [26], the Efficacy of Nitric Oxide in Stroke (ENOS) trial testing transdermal glyceryl trinitrate [27], the China Antihypertensive Trial in Acute Ischaemic Stroke (CATIS) that was focused on ischaemic stroke patients not receiving intravenous thrombolysis and investigated different anti-hypertensive regimens [28], the Prevention Regime for Effectively Avoiding Second Strokes (PRoFESS) trial testing telmisartan [29], all showed that BP lowering had no effect on functional outcomes post-stroke. In contrast, patients with an acute (<24 h) anterior circulation stroke treated with nimodipine during the Intravenous Nimodipine West European Stroke (INWEST) trial were shown to present worse functional outcomes at 21 days compared to placebo, especially in the subgroup of patients with substantial (>20%) diastolic blood pressure decrease from baseline [30].

On the other hand, pharmacologically induced hypertension may also be needed, especially in patients with fluctuating symptoms and hypotension. The rationalisation of such a therapeutic option may have the potential to maintain adequate BP levels, enhancing collateral circulation and preserving or augmenting cerebral perfusion. To that aim, the Safety and Efficacy of Therapeutic Induced Hypertension in Acute Non-Cardioembolic Ischaemic Stroke (SETIN-HYPERTENSION) trial included patients with acute progressive, non-cardioembolic stroke, ineligible for reperfusion therapies and randomised them in receiving phenylephrine-induced hypertension versus standard of care [31]. In this trial, drug-induced hypertension was associated with higher odds of early neurological improvement; however, 3-month functional outcomes were similar compared to placebo.

All of the above clinical trials have consistently overlooked the role of BPV in this setting. During the hyperacute phase of ischaemic stroke, BPV has demonstrated an independent and linear association with early neurological deterioration during hospitalisation [32,33]. Furthermore, it was also correlated with 90-day mortality, independently of the mean levels of systolic and diastolic BP [34,35]. Additionally, BPV was shown to be independently associated with increased infarct growth and an increased likelihood of parenchymal haemorrhage [36,37]. According to a systematic review and meta-analysis of 18 observational studies, systolic BPV was also significantly associated with poor functional outcomes and dependency at 3 months [11]. A more recent, updated systematic review and meta-analysis, that included 34 observational studies, confirmed that higher BPV is associated with worse functional outcomes, mortality, and early neurological deterioration among acute ischaemic stroke patients [38]. However, whether there is a direct causal relationship between BPV and adverse outcomes post-stroke is still debatable [39,40]. Reverse causality (i.e., adverse outcomes leading to increased BPV as a physiological response) and the possibility of BPV being merely an epiphenomenon (as a marker of underlying vascular dysfunction) are significant factors that complicate the interpretation of observational studies.

Randomised controlled clinical trial data regarding BPV for ischaemic stroke patients not eligible for reperfusion therapies are provided through a post-hoc analysis of the Controlling Hypertension After Severe Cerebrovascular Event (CHASE) trial [41]. Patients with severe ischaemic stroke presenting within the first 72 h after symptom onset were included in the CHASE trial and were randomised when receiving a more individualised BP-lowering approach versus the standard one. After enrolling 483 patients, no difference was shown between the two groups regarding poor functional outcomes. Apart from the main trial results, a post-hoc analysis was undertaken, including 442 patients in the acute phase (during the first day post-randomisation) and 390 patients in the subacute phase (between the second and seventh day post-randomisation) that had available BP data during the whole monitoring timing, allowing for the calculation of several BPV indices: mean, maximum, minimum, standard deviation, coefficient of variation, successive variation and average real variability, and functional successive variation as the key indicator [42]. According to this analysis, six measurements of BPV during the subacute phase rather than the acute phase were strongly correlated with a 90-day mRS of 3 or more. The finding that subacute rather acute BPV may be associated with worse functional outcomes is further confirmed by other studies as well, including the analysis of the Fukuoka Stroke Registry [43], the post-hoc analysis of the Controlling Hypertension and Hypotension Immediately Post-Stroke (CHHIPS) and the Continue or Stop Post-Stroke Antihypertensives Collaborative Study (COSSACS) trials [44], and the post-hoc analysis of the Head Positioning in Acute Stroke Trial (HeadPoST) trial [45]. Judging by these findings, it seems that BPV over very short durations does not alter post-stroke outcomes, while a more longstanding BPV (over 24 h or longer) may be needed to have an actual effect on stroke outcomes, at least for the patients that do not receive acute reperfusion therapies. This fact should be taken into account during the design of future randomised controlled clinical trials, allowing for longer BPV recordings.

One small, single-center, double-blind, randomised clinical trial, that was conducted in Korea, analysed 62 patients with acute ischaemic stroke receiving fimasartan versus valsartan with the aim of comparing BPV between the two treatment regimens [46]. According to the trial’s results, fimasartan showed a greater effect on reducing BPV as measured at 8 weeks compared to valsartan. However, the applicability of these results in the acute setting is questionable, considering that BPV was measured at 8 weeks after randomisation and that patients could be included in the study within 7 days of stroke onset, missing a potentially determining time window for intervention. Additionally, this study provided no assessment of clinical outcomes among the two groups. Similarly, another open-label feasibility study aimed to enroll 150 patients within 7 days of ischaemic stroke, comparing the effects of two different antihypertensive drug classes (calcium channel or angiotensin-converting enzyme inhibitor/angiotensin receptor blocker regime) on BPV, as measured at day 21 and at day 90 [47]. Importantly, this study had prespecified a modified Rankin Scale score as a primary exploratory outcome. However, this study managed to recruit only 14 patients, five of whom were lost to follow-up, rendering comparison between the treatment regimens unfeasible [48].

## 5. Blood Pressure Variability among Patients with Acute Ischaemic Stroke Receiving Intravenous Thrombolysis

According to both the American Heart Association/American Stroke Association and the European Stroke Organisation guidelines, BP levels should be maintained <185/110 mmHg before and <180/105 mmHg during intravenous thrombolysis for acute ischaemic stroke [6,7]. Targeting BP levels even lower than <180/105 mmHg was suggested by several observational studies and meta-analyses, with the notion that lower targets may be related to higher odds of clinical improvement and a lower risk of symptomatic intracranial haemorrhage [49,50,51,52]. However, caution is warranted when a more aggressive BP-lowering protocol is considered, as critically low BP levels may jeopardise penumbral tissue perfusion and lead to the expansion of cerebral infarction [53]. The Enhanced Control of Hypertension and Thrombolysis Stroke Study (ENCHANTED) trial, using a quasi-factorial, open-label design, aimed to investigate the efficacy and safety of more aggressive BP reduction (systolic BP target of 130–140 mmHg) in a randomised setting [54]. After enrolling 2196 patients eligible for intravenous thrombolysis, no difference was demonstrated regarding the functional outcomes at 3 months between aggressively and conservatively treated patients, despite the fact that there were fewer intracranial haemorrhages in the interventional arm. It should be noted, though, that the ENCHANTED trial was limited by several factors: small final difference in BP levels between the two groups, open-label design, low generalisability and inclusion of patients whose initial BP levels were already within the recommended target [55]. On top of those shortcomings, BPV was also not included as a variable of interest.

In a post-hoc analysis of the Efficacy and Safety of magnetic resonance imaging-based Thrombolysis in Wake-Up Stroke (WAKE-UP) trial [56], although mean systolic BP was associated with a smaller treatment effect of alteplase, there was no correlation of BPV (as expressed by the coefficient of variation) with the outcomes of post-intravenous thrombolysis [57]. Similarly, a systematic review and meta-analysis of observational studies showed that BPV may be associated with worse functional outcomes after endovascular treatment but there is no clear association between BPV and functional outcomes (RR = 1.08; 95%CI 0.96–1.22) or symptomatic intracranial haemorrhage (RR = 2.40; 95%CI 0.71–8.03) after intravenous thrombolysis [12]. However, as acknowledged by the authors, the analysis regarding intravenous thrombolysis was rather limited by the restricted number of included studies (*n* = 3). In fact, other studies have shown that the efficacy and safety of acute recanalisation therapies may indeed be influenced by BPV. Early BPV was shown to be associated with poor outcomes at 3 months [58], lower odds of reduced disability (shift analysis) [59], greater diffusion-weighted imaging lesion growth [60], mortality [61], greater risk for symptomatic intracerebral haemorrhage after intravenous thrombolysis in acute ischaemic stroke [61,62,63,64] and recurrent stroke at 3 months [65]. Notably, Malhorta and colleagues reported in a recent meta-analysis that elevated post-treatment systolic BPV levels (quantified by successive variation) were observed in patients with symptomatic intracranial haemorrhage [52]. Finally, a post-hoc analysis of the Combined Lysis of Thrombus using Ultrasound and Systemic Tissue Plasminogen Activator for Emergent Revascularisation (CLOTBUST-ER trial) documented that pulse pressure variability was identified as the BP parameter with the most parsimonious fit in multivariable models of all key stroke outcomes, and was independently associated with a lower likelihood of both 24 h neurological improvement and 90-day independent functional outcomes [66]. Additionally, pulse pressure variability was also independently related to increased odds of any intracranial bleeding and 90-day mortality [66].

On the other hand, BPV did not appear to be associated with early neurological deterioration specifically among patients with small subcortical infarcts treated with intravenous thrombolysis [67]. Similarly, in another more fragile patient subgroup, those with symptomatic intracranial artery stenosis or occlusion, BPV, as expressed by standard deviation, was again not associated with early neurological deterioration, although there was a clear negative association with 3-month functional outcomes [68].

Although it seems that BPV has a significant effect on the outcomes of patients treated with intravenous thrombolysis, there are no randomised controlled clinical trial data to further address this potential association. It would be of value to assess BPV at least as a secondary analysis in future trials investigating BP management in the setting of intravenous thrombolysis administration.

## 6. Blood Pressure Variability among Patients with Acute Ischaemic Stroke Receiving Endovascular Treatment

There is an increasing interest regarding BP management in the setting of endovascular treatment for acute ischaemic stroke. Not only should high BP levels be promptly treated (pre-thrombectomy levels ≤ 185/110 mmHg and during thrombectomy ≤ 180/105 mmHg) [6,7], since they are related to an unfavourable prognosis and symptomatic intracranial haemorrhage [69,70,71,72], but also hypotension should be avoided since it was independently associated with infarct expansion and worse functional outcomes at 3 months [73,74,75,76]. In fact, a U-shaped association was demonstrated between mean systolic BP levels and functional outcomes after endovascular therapy, according to an individual patient data meta-analysis that included data from three randomised controlled clinical trials comparing general versus local anesthesia among patients treated with mechanical thrombectomy [77].

Apart from targeting a more restricted BP range (target mean-BP levels between 70 and 90 mmHg) [77], it seems that there is an additional need to further individualise BP management. Recanalisation status is one of the most important confounders affecting BP management and outcomes: lower than the currently recommended BP levels (i.e., targeting systolic BP levels lower than 140 mmHg) following successful recanalisation was associated with favourable functional outcomes, as shown in several observational studies [75,76,78]. For that reason, six randomised controlled clinical trials were conducted with the aim of answering whether a more intensive BP management plan versus the conventional one may lead to better outcomes among patients achieving successful recanalisation [79,80,81,82,83,84]. However, according to those trials’ results, stricter BP control was not associated with better clinical outcomes or lower odds of symptomatic intracranial haemorrhage [79,80,81,82,83]. On the contrary, higher odds of poor functional outcomes were demonstrated among patients receiving intensive BP management, which led to the premature termination of two randomised controlled clinical trials [80,81].

A potential explanation for the lack of success of those randomised controlled clinical trials could be the possibility of reverse causality between BP levels and outcomes post-endovascular treatment, i.e., high BP levels per se do not lead to worse outcomes but compensate against poor cerebral perfusion. Another reason could be the failure to consider BPV during BP management. In fact, according to a post-hoc analysis of Blood Pressure Target in Acute Ischaemic Stroke to Reduce Haemorrhage After Endovascular Therapy (BP TARGET) trial [79], it was shown that BPV was significantly higher in the intensive BP management group, although it did not affect the outcomes among the 290 included patients [85]. However, according to an individual patient data meta-analysis that included 2460 patient data derived from five observational studies, systolic BPV within the first 24 h post-EVT, as expressed by the highest tertile of both standard deviation and coefficient of variation, was associated with higher mortality and disability at 3 months, independently of the mean systolic BP levels [86]. These results were further confirmed by several other cohort studies during the last few years (Table 2) [87,88,89,90], highlighting that the association of BPV with adverse clinical outcomes may even be intensified among patients receiving general anesthesia [91] or additional rescue treatment with balloon angioplasty or stenting [92]. One study-level meta-analysis that included 11 studies comprising more than 3500 patients, confirmed that higher systolic BPV was associated with lower odds of achieving good functional outcomes at 3 months [93].

Interestingly, the majority of the studies in this field reject the hypothesis that BPV is associated with symptomatic intracranial haemorrhage post-endovascular treatment [86,93,97,104]. Thus, the independent association of BPV with the clinical outcomes might be better explained by sustained hypoperfusion despite recanalisation [105,106], arguing against intensive BP reduction in this setting. In any case, given the strong association of BPV with short-term and long-term outcomes post-endovascular treatment [102,103,107], future research designs that incorporate BPV as a target within the BP management protocol are warranted. However, a key question arises: how can patients at risk of developing increased BPV be identified and effectively managed? Numerous clinical prediction models for BPV were proposed, yet their prognostic ability remains limited [88]. Therefore, there is a need for further optimisation, potentially leveraging artificial intelligence and machine learning techniques [108,109]. Additionally, spectral analysis for rapid BPV assessment shows promise in promptly identifying at-risk patients without requiring lengthy BP recordings [110]. After identifying patients at risk, imaging techniques, such as the assessment of the collateral status [111,112,113] and the degree of recanalisation post-endovascular treatment [114,115] or the evaluation of the dynamic brain autoregulation in real-time using near-infrared spectroscopy-derived tissue oxygenation [116] or transcranial doppler [117], could guide BP management within targeted and tighter ranges to prevent increased systolic BPV.

In terms of antihypertensive treatment, calcium channel blockers and non-loop diuretic drugs, either alone or as adjunctive to other agents, were shown to reduce BPV, while beta-blockers were associated with elevated BPV [118,119]. Apart from affecting BPV, antihypertensive treatment may also interact with the association of BPV with outcomes by ameliorating its effect on adverse functional outcomes post-endovascular treatment, as indicated by a small, retrospective study [120]. Furthermore, it is prudent to avoid increasing iatrogenic BPV by limiting the use of potent, short-acting antihypertensive drugs and promoting adherence to previous medications whenever possible [7,121]. Similarly, hypotension should be avoided, and volume restoration may be considered in depleted patients, guided by BPV indices. Finally, atorvastatin was previously associated with a decrease in BPV among individuals with hypertension, as demonstrated in the outpatient setting rather than during acute stroke care [122]. Hence, it is essential to further assess the potential impact of statins, either alone or in combination with antihypertensive medications, on BPV, specifically during acute stroke management.

## 7. Blood Pressure Variability in Patients with Intracerebral Haemorrhage

During the last decade, research interest has actively turned towards the management of intracerebral haemorrhage. However, several randomised controlled clinical trials investigating different haemostatic factors or neurosurgical approaches to restrict haematoma expansion have failed to provide the anticipated results for better outcomes in spontaneous intracerebral haemorrhage. Therefore, the optimisation of the more conservative interventions, including BP control, seems to be the cornerstone for the management of intracerebral haemorrhage [123]. Indeed, the third Intensive Care Bundle with Blood Pressure Reduction in Acute Cerebral Haemorrhage Trial (INTERACT3), that included patients with intracerebral haemorrhage within the first 6 h post-onset, showed that the implementation of an acute (within the first hour after admission to the hospital) care bundle protocol that consisted of BP lowering, correction of hyperglycemia and hyperpyrexia and reversal of anticoagulation when needed, was associated with better clinical outcomes [124]. In fact, intravenous BP-lowering treatments were the most common intervention used in the INTERACT3, administered in almost 80% of the patients during the first 24 h [124]. The results of the INTERACT3 came to further confirm the previously conducted Intensive Blood Pressure Reduction in Acute Cerebral Haemorrhage Trial 2 (INTERACT2) that, although missing the primary endpoint (death or disability at 3 months), showed that patients receiving intensive BP reduction had improved functional outcomes according to the ordinal analysis of the modified Rankin Scale score at 3 months [125]. Importantly, a post-hoc analysis of the INTERACT2 was conducted to specifically evaluate the effect of BPV on the outcomes of patients with intracerebral haemorrhage treated either intensively or conservatively [126]. According to this analysis, BPV, as evaluated both in the hyperacute (within the first 24 h) and the acute phase (between days 2 and 7), emerged as an independent predictor of mortality and disability at 3 months, underscoring the need for a more cautious BP reduction to avoid increased BPV [126]. That is also captured by the European Stroke Guidelines on blood pressure management: the decrease in systolic BP should not exceed 90 mmHg from baseline values, according to an expert consensus statement [7].

The adverse effects of BPV among patients with acute intracerebral haemorrhage were confirmed by other studies as well. Systolic BPV was independently associated with worse short-term [127,128,129,130] and long-term outcomes (Table 3) [23,45,128,129,131,132,133,134,135]. A systematic review and meta-analysis summarised the findings of seven of those studies and provided conclusive evidence that BPV is associated with a higher risk of poor functional outcomes at 3 months [136]. Furthermore, an individual patient data meta-analysis was also conducted, including patient data from the INTERACT2 and the Antihypertensive Treatment of Acute Cerebral Haemorrhage II (ATACH-II) trials, and supported the prognostic significance of BPV for mortality at 3 months [137]. Interestingly, in the majority of the studies, BPV was not associated with haematoma expansion [126,127,128,132], despite the fact that earlier reports raised concerns about the relationship between increased BPV and the risk of haematoma growth [130]. Indeed, an analysis of the FAST-MAG trial, that was dedicated to this questionable association, confirmed that BPV was not related to the occurrence of haematoma expansion [24].

Based on those observations, it seems that haematoma expansion is not the prevailing mechanism through which BPV may lead to worse clinical outcomes [138], despite the fact that sudden BP rises were associated with haematoma enlargement [130,139]. Other alternative, pathophysiological mechanisms could be the promotion of perihematomal edema due to increased BP variations [140,141], or the hypoperfusion of perihematomal tissues in an already impaired brain with disrupted autoregulatory capacities [142,143]. Additionally, as indicated by the fact that very short-term BPV (such as the beat-to-beat BPV, which is mostly regulated by the autonomic nervous system) may also be associated with worse outcomes in intracerebral haemorrhage [133], autonomic dysregulation may also have a potential role through subsequent inflammatory changes and endothelial dysfunction [144,145].

Despite the underlying pathophysiological mechanisms, it seems that BPV should be considered in BP management among patients with intracerebral haemorrhage. “Time is brain” is irrefutable for both ischaemic and haemorrhagic stroke [146]. The investigators of the Evaluation of Patients with Acute Hypertension and Intracerebral Haemorrhage with Intravenous Clevidipine Treatment (ACCELERATE) trial reported that clevidipine monotherapy was effective and safe for rapid BP reduction in this cohort of critically ill patients with intracerebral haemorrhage [147]. The median time to achieve the systolic BP target range was 5.5 min and all patients achieved the target systolic BP within 30 min [147]. However, clinicians should respect the delicate equilibrium of cerebral perfusion and avoid very sudden drops of BP in the cases of intracerebral haemorrhage. Preliminary evidence from a single-center study suggests that nicardipine might be a safe antihypertensive medication to reduce BP in the setting of spontaneous intracerebral haemorrhage, while at the same time reducing BPV when administered alone or in combination with labetalol or hydralazine [148].

## 8. Blood Pressure Variability in Patients with Subarachnoid Haemorrhage

Outside the setting of primary intracranial haemorrhage, one single-center study conducted in China evaluated BPV among 612 patients with acute aneurysmal subarachnoid haemorrhage, showing that BPV, as expressed by the standard deviation and the successive variation of the systolic BP levels, was associated with rebleeding [149]. Higher systolic BPV was also associated with higher odds of worse outcomes among 303 patients with subarachnoid haemorrhage, as defined by the Glasgow Outcome Scale scores of 1–3 at discharge [150]. Similar results were confirmed by more studies in this setting [151,152]. Noteworthy, increased visit-to-visit BPV was also correlated with the growth of unruptured intracranial aneurysms [153]. Therefore, clinicians should consider BPV during the management of intracranial aneurysms and subarachnoid haemorrhage as well.

## 9. Conclusions

In conclusion, the management of BPV in the context of acute stroke represents a multifaceted challenge with significant implications for patient outcomes. As highlighted throughout this narrative review, BPV emerges as a critical factor influencing the prognosis of patients across various stages of acute stroke care. In the prehospital setting, while current guidelines lack specific recommendations for BP management, emerging evidence suggests that BPV could play a role in predicting outcomes, albeit limited by methodological constraints. Similarly, among acute ischaemic stroke patients who are ineligible for reperfusion therapies, BPV appears to independently impact functional outcomes, emphasising the need for tailored approaches to BP control. During intravenous thrombolysis and endovascular treatment, the intricate balance between BP levels, recanalisation status and BPV becomes apparent. While aggressive BP lowering may mitigate intracranial haemorrhage risk, it should be balanced against potential hypoperfusion risks associated with increased BPV. Furthermore, intracerebral haemorrhage presents a unique challenge where BPV is increasingly recognised as a predictor of mortality and disability. Striking a delicate balance between reducing haematoma expansion and avoiding hypoperfusion-related complications underscores the importance of nuanced BP management strategies. Finally, studies in the setting of acute subarachnoid haemorrhage indicate a correlation of BPV with rebleeding risk and worse outcomes, emphasising the need for BPV monitoring in this population. Integrating BPV assessment into clinical practice and research protocols is imperative for refining treatment strategies and improving outcomes in acute stroke care. Future studies should explore novel interventions targeting BPV modulation and leverage advanced monitoring techniques to optimise BP management tailored to individual patient needs.

## Figures and Tables

**Table 1:** Blood pressure variability indices commonly used in stroke trials.

Index	Description	Formula
Standard Deviation (SD)—mmHg	Denotes the dispersion of BP measurements around the mean. It is more resilient to the influence of isolated extreme values compared to the maximum–minimum range, given a sufficient number of measurements. Disadvantages include its correlation with average BP levels and susceptibility to BP trends, especially in 24 h recordings due to diurnal BP variations.	$\sqrt{\frac{1}{\left(n-1\right)}\sum_{\left(i=1\right)}^{\left(n\right)}{\left(BPi-BPmean\right)}^{2}}$
Coefficient of Variation (CV)—%	The ratio of SD to the mean BP. While it can mitigate the correlation between SD and average BP levels, it still remains susceptible to BP trends.	$\frac{SD\;BP}{BP\;mean}\times 100$
Weighted 24 h SD—mmHg	Adjusts for the nocturnal BP lowering and its impact on the 24 h SD by factoring in a weighted average of the daytime and nighttime SD. it may still be influenced by trends occurring within the daytime and nighttime periods.	$\sqrt{{\left(\frac{n_{day}}{n_{total}}{SD}_{day}\right)}^{2}+{\left(\frac{n_{night}}{n_{total}}{SD}_{night}\right)}^{2}}$
Successive Variation (SV)—mmHg	Refers to the fluctuation or change observed between consecutive blood pressure readings over a given period of time and provides valuable information about the short-term variability of blood pressure. Susceptible to BP trends.	$\sqrt{\frac{1}{\left(n-1\right)}\sum_{\left(i=1\right)}^{\left(n-1\right)}{\left({BP}_{i+1}-{BP}_{i}\right)}^{2}}$
Residual Variability (RV)—mmHg [2]	A 24 h total BP variance by using spectral analysis and removing first and second harmonics to exclude circadian changes	$\sum_{i=1}^{n}{\left({BP}_{i}-CC\right)}^{2}$
		where *CC* corresponds to the cycling components of the circadian BP pattern.
Average Real Variability (ARV)—mmHg	Overall variability of differences between successive readings over 24 h or over different days. Resilient to BP trends but affected by poor data quality and missing values.	$\frac{1}{n-1}\sum_{i=1}^{n-1}\left|{BP}_{i+1}-{BP}_{i}\right|$
Variation Independent of Mean (VIM)—mmHg	A more sophisticated modification of SD that results through nonlinear regression to eliminate its correlation with average BP.	$\frac{SD}{{BP}^{x}}\times {\left[\bar{BP}\right]}^{x}$
		where power *x* is computed as fitting a curve through nonlinear regression.
Time Rate (TR)—mmHg/min	Capable of determining not only the grade of BPV but also the speed of BP fluctuations. May be limited by fixed intervals between measurements, such as in ambulatory blood pressure monitoring.	$\frac{1}{n-1}\sum_{i=1}^{n-1}\frac{\left|{BP}_{i+1}-{BP}_{i}\right|}{t_{i+1}-t_{i}}$
Mean Absolute Change (MAC)—mmHg	Used as a quantitative index of variability which is less dependent on the mean BP value.	$MAC=\frac{\left|\Delta BP\right|}{\Delta Time}$
Range—mmHg	Heavily influenced by outliers.	$Max\left({BP}_{i}\right)-min\left({BP}_{i}\right)$

**Table 2:** Available studies investigating the potential association of blood pressure variability and outcomes among patients with acute ischaemic stroke receiving endovascular treatment.

Study	Design	Period of Enrollment	Patients	BPV Measurement	BPV Indices	Results	
						Adverse Functional Outcomes	Symptomatic Intracranial Haemorrhage
BP TARGET [85]	Post-hoc analysis of RCT	2017–2019	290	34 BP measurements within the first 24 h	SD, CV, range, SV, TR	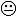	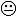
	BPV was not associated with functional outcomes. BPV was not associated with symptomatic intracranial haemorrhage.						
Palaiodimou et al. [86]	Individual patient data meta-analysis from 5 observational studies [94,95,96,97,98]	2012–2016 [94] 2013–2017 [95] 2014–2017 [96] 2011–2016 [97] 2005–2015 [98]	2460	At least 4 BP measurements within the first 24 h	SD, CV	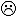	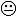
	BPV was associated with higher mortality, death or disability and functional impairment at 3 months. BPV was not associated with symptomatic intracranial haemorrhage.						
Kim et al. [99]	Retrospective	2013–2017	211	24 BP measurements within the first 24 h	Range, SD, CV, SV, TR	NR	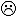
	BPV was associated with symptomatic intracranial haemorrhage.						
Anadani et al. [100]	Retrospective	2014–2017	298	24 BP measurements within the first 24 h	SD, CV, range	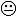	NR
	BPV was not associated with functional outcomes.						
Chu et al. [101]	Retrospective	2014–2018	224	24 BP measurements within the first 24 h	SD	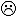	NR
	BPV was associated with worse functional outcomes at 3 months.						
Mistry et al. [88]	Post-hoc analysis of a prospective, observational study	2017–2018	443	Within the first 24 h.	SD, CV, SV, RSD, ARV	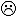	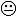
	BPV was associated with poor functional outcomes or death at 3 months. BPV was not associated with symptomatic intracranial haemorrhage.						
Zhang et al. [90]	Retrospective	2015–2018	72	24 BP measurements within the first 24 h	SD, CV, SV	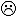	NR
	BPV was associated with worse functional outcomes at 3 months.						
Zhou et al. [102]	Retrospective	2016–2019	95	24 BP measurements within the first 24 and 48 h	IQR	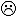	NR
	BPV was associated with worse functional outcomes at discharge.						
Prasad et al. [103]	Retrospective	2012–2019	2041	50 BP measurements within the first 72 h	SD, CV, ARV, SV, RSD	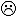	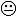
	BPV was associated with worse functional outcomes and mortality at 3 months. BPV was not associated with haemorrhagic transformation or symptomatic intracranial haemorrhage.						
Yang et al. [87]	Retrospective	2015–2020	257	12 BP measurements within the first 24 h	PPV, SD, SV, range	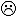	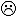
	BPV was associated with worse functional outcomes at 3 months. BPV was associated with symptomatic intracranial haemorrhage.						
Qin et al. [89]	Retrospective	NR	236	Hyperacute: 8 BP measurements within the first 2 h Acute: 22 BP measurements between 2 and 24 h Subacute: 6 BP measurements between 2 and 7 days.	SD, CV, ARV, range	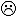	NR
	BPV was associated with worse functional outcomes at 3 months.						
Xu et al. [91]	Prospective	2018–2020	141	Every 5 min during endovascular treatment	SD, CV, range, SV	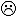	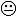
	BPV was associated with worse functional outcomes at 3 months. BPV was not associated with parenchymal haemorrhage.						
Lu et al. [92]	Retrospective	2017–2021	458	24 BP measurements within the first 24 h	SD	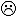	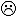
	BPV was associated with worse functional outcomes at 3 months. BPV was associated with mortality at 30 days. BPV was associated with symptomatic intracranial haemorrhage.						

BPV: blood pressure variability; RCT: randomised controlled clinical trials; SD: standard deviation; CV: coefficient of variation; SV: successive variation; PPV: pulse pressure variability; ARV: average real variability; IQR: interquartile range; TR: time rate; RSD: residual standard deviation; NR: not reported; 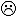: association with adverse outcomes; 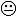: no association.

**Table 3:** Available studies investigating the potential association of blood pressure variability and outcomes among patients with intracerebral haemorrhage.

Study	Design	Period of Enrollment	Patients	BPV Measurement	BPV Indices	Results	
						Adverse Clinical Outcomes	Haematoma Expansion
INTERACT2 [126]	Post-hoc analysis of RCT	2008–2012	2645	Hyperacute phase: 5 BP measurements within the first 24 h Acute phase: 12 measurements taken on days 2–7	SD, CV, VIM, RSD, ARV	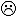	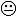
	BPV in both the hyperacute and acute phases was associated with death/major disability and worse functional outcomes at 3 months. BPV was not associated with haematoma expansion.						
FAST-MAG [23,24]	Post-hoc analysis of RCT	2005–2012	386	11 BP measurements within the first 24 h	SD, CV, SV	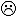	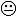
	BPV was associated with poor functional outcomes at 3 months. BPV was not associated with haematoma expansion.						
ATACH-2 [132]	Post-hoc analysis of RCT	2011–2015	913 (acute phase) 877 (subacute phase)	Acute phase: 44 BP measurements within the first 24 h Subacute phase: 12 BP measurements taken on days 2–7	SD, CV, SV, ARV, RSD	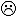	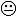
	BPV in both the acute and subacute phases was associated with poor functional outcomes at 3 months. BPV was not associated with haematoma expansion.						
HeadPoST [45]	Post-hoc analysis of RCT	2015–2016	817	6 BP measurements within the first 24 h	CV	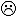	NR
	BPV was associated with an unfavourable shift of modified Rankin Scale score at 3 months. BPV was associated with poor functional outcomes at 3 months.						
Rodriguez-Luna et al. [130]	Prospective	2009–2010	117	96 BP measurements within the first 24 h	SD, maximum BP increase from baseline, minimum BP and maximum BP drop from baseline values, percentage of 24 h BP monitoring values exceeding 180 and 130 mmHg	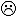	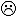
	BPV was associated with haematoma growth at 24 h. BPV was associated with early neurological deterioration at 24 h.						
SAMURAI-ICH [128]	Prospective	2009–2011	205	24 BP measurements within the first 24 h	SD, SV	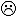	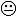
	BPV was associated with neurological deterioration at 72 h and with an unfavourable functional outcome at 3 months. BPV was not associated with haematoma expansion.						
Lattanzi et al. [131]	Retrospective	2007–2013	138	18 BP measurements within the first 72 h	SD, CV, maximum–minimum difference	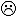	NR
	BPV was associated with poor functional outcomes at 3 months.						
Divani et al. [127]	Retrospective	2008–2017	762	16 BP measurements within the first 24 h	SD, CV, SV, range, functional SV	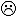	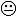
	BPV was associated with death and disability at discharge. BPV was not associated with haematoma expansion.						
Jeon et al. [135]	Retrospective	2008–2016	104	23 BP measurements within the first 24–48 h	SD, CV, MAC, range	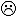	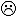
	BPV was associated with poor functional outcomes higher shift of modified Rankin Scale score at 3 months. MAC was associated with haematoma growth.						
Zhang et al. [134]	Prospective	2013–2016	131	32 BP measurements within the first 24 h	SD, CV, range	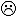	NR
	BPV was associated with poor functional outcomes at 3 months.						
Meeks et al. [129]	Prospective	2016–2018	566	NR	SD	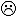	NR
	BPV was associated with poor functional outcomes at discharge, at 1 and at 3 months. BPV was associated with mortality during hospitalisation, at 1 and at 3 months.						
Qu et al. [133]	Prospective	2014–2020	66	Continuously for 5 min for calculation of beat-to-beat BPV	SD, VIM	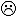	NR
	Beat-to-beat BPV within 1–2 days after stroke onset was associated with unfavourable outcomes at 3 months.						

BPV: blood pressure variability; RCT: randomised controlled clinical trials; SD: standard deviation; CV: coefficient of variation; VIM: variation independent of mean; RSD: residual standard deviation; ARV: average real variability; SV: successive variation; MAC: mean absolute change; NR: not reported; 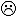: association with adverse outcomes; 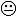: no association.

## References

[1] Li R.C., Xu W.D., Lei Y.L., Bao T., Yang H.W., Huang W.X., Tang H.R. (2019). The risk of stroke and associated risk factors in a health examination population: A cross-sectional study. Medicine.

[2] Wallace J.D., Levy L.L. (1981). Blood pressure after stroke. JAMA.

[3] Carlberg B., Asplund K., Hägg E. (1991). Factors influencing admission blood pressure levels in patients with acute stroke. Stroke.

[4] Willmot M., Leonardi-Bee J., Bath P.M. (2004). High blood pressure in acute stroke and subsequent outcome: A systematic review. Hypertension.

[5] Vemmos K.N., Tsivgoulis G., Spengos K., Zakopoulos N., Synetos A., Manios E., Konstantopoulou P., Mavrikakis M. (2004). U-shaped relationship between mortality and admission blood pressure in patients with acute stroke. J. Intern. Med..

[6] Powers W.J., Rabinstein A.A., Ackerson T., Adeoye O.M., Bambakidis N.C., Becker K., Biller J., Brown M., Demaerschalk B.M., Hoh B. (2019). Guidelines for the Early Management of Patients with Acute Ischemic Stroke: 2019 Update to the 2018 Guidelines for the Early Management of Acute Ischemic Stroke: A Guideline for Healthcare Professionals from the American Heart Association/American Stroke Association. Stroke.

[7] Sandset E.C., Anderson C.S., Bath P.M., Christensen H., Fischer U., Gąsecki D., Lal A., Manning L.S., Sacco S., Steiner T. (2021). European Stroke Organisation (ESO) guidelines on blood pressure management in acute ischaemic stroke and intracerebral haemorrhage. Eur. Stroke J..

[8] Parati G., Bilo G., Kollias A., Pengo M., Ochoa J.E., Castiglioni P., Stergiou G.S., Mancia G., Asayama K., Asmar R. (2023). Blood pressure variability: Methodological aspects, clinical relevance and practical indications for management—A European Society of Hypertension position paper. J. Hypertens..

[9] Parati G., Stergiou G.S., Dolan E., Bilo G. (2018). Blood pressure variability: Clinical relevance and application. J. Clin. Hypertens..

[10] Dawson S.L., Panerai R.B., Potter J.F. (2003). Serial changes in static and dynamic cerebral autoregulation after acute ischaemic stroke. Cerebrovasc. Dis..

[11] Manning L.S., Rothwell P.M., Potter J.F., Robinson T.G. (2015). Prognostic Significance of Short-Term Blood Pressure Variability in Acute Stroke: Systematic Review. Stroke.

[12] Qin J., Zhang Z. (2020). Prognostic significance of early systolic blood pressure variability after endovascular thrombectomy and intravenous thrombolysis in acute ischemic stroke: A systematic review and meta-analysis. Brain Behav..

[13] Tsivgoulis G., Ntaios G. (2012). Blood pressure variability in subacute ischemic stroke: A neglected potential therapeutic target. Neurology.

[14] Hawkes M.A., Anderson C.S., Rabinstein A.A. (2022). Blood Pressure Variability After Cerebrovascular Events: A Possible New Therapeutic Target: A Narrative Review. Neurology.

[15] Kobayashi A., Czlonkowska A., Ford G.A., Fonseca A.C., Luijckx G.J., Korv J., de la Ossa N.P., Price C., Russell D., Tsiskaridze A. (2018). European Academy of Neurology and European Stroke Organization consensus statement and practical guidance for pre-hospital management of stroke. Eur. J. Neurol..

[16] Ankolekar S., Fuller M., Cross I., Renton C., Cox P., Sprigg N., Siriwardena A.N., Bath P.M. (2013). Feasibility of an ambulance-based stroke trial, and safety of glyceryl trinitrate in ultra-acute stroke: The rapid intervention with glyceryl trinitrate in Hypertensive Stroke Trial (RIGHT, ISRCTN66434824). Stroke.

[17] RIGHT-2 Investigators (2019). Prehospital transdermal glyceryl trinitrate in patients with ultra-acute presumed stroke (RIGHT-2): An ambulance-based, randomised, sham-controlled, blinded, phase 3 trial. Lancet.

[18] van den Berg S.A., Uniken Venema S.M., Reinink H., Hofmeijer J., Schonewille W.J., Miedema I., Fransen P.S.S., DM O.P., Raaijmakers T.W.M., van Dijk G.W. (2022). Prehospital transdermal glyceryl trinitrate in patients with presumed acute stroke (MR ASAP): An ambulance-based, multicentre, randomised, open-label, blinded endpoint, phase 3 trial. Lancet Neurol..

[19] Shaw L., Price C., McLure S., Howel D., McColl E., Younger P., Ford G.A. (2014). Paramedic Initiated Lisinopril For Acute Stroke Treatment (PIL-FAST): Results from the pilot randomised controlled trial. Emerg. Med. J..

[20] Saver J.L., Starkman S., Eckstein M., Stratton S.J., Pratt F.D., Hamilton S., Conwit R., Liebeskind D.S., Sung G., Kramer I. (2015). Prehospital use of magnesium sulfate as neuroprotection in acute stroke. N. Engl. J. Med..

[21] Gioia L.C., Zewude R.T., Kate M.P., Liss K., Rowe B.H., Buck B., Jeerakathil T., Butcher K. (2016). Prehospital systolic blood pressure is higher in acute stroke compared with stroke mimics. Neurology.

[22] Shriki J., Johnson L., Patel P., McGann M., Lurie T., Phipps M.S., Yarbrough K., Jindal G., Mubariz H., Galvagno S.M. (2020). Transport Blood Pressures and Outcomes in Stroke Patients Requiring Thrombectomy. Air Med. J..

[23] Chung P.W., Kim J.T., Sanossian N., Starkmann S., Hamilton S., Gornbein J., Conwit R., Eckstein M., Pratt F., Stratton S. (2018). Association Between Hyperacute Stage Blood Pressure Variability and Outcome in Patients with Spontaneous Intracerebral Hemorrhage. Stroke.

[24] Oh D.M., Shkirkova K., Poblete R.A., Chung P.W., Saver J.L., Starkman S., Liebeskind D.S., Hamilton S., Wilson M., Sanossian N. (2023). Association Between Hyperacute Blood Pressure Variability and Hematoma Expansion after Intracerebral Hemorrhage: Secondary Analysis of the FAST-MAG Database. Neurocrit Care.

[25] Song L., Chen C., Chen X., Guo Y., Liu F., Lin Y., Billot L., Li Q., Liu H., Si L. (2021). INTEnsive ambulance-delivered blood pressure Reduction in hyper-ACute stroke Trial (INTERACT4): Study protocol for a randomized controlled trial. Trials.

[26] Sandset E.C., Bath P.M., Boysen G., Jatuzis D., Kõrv J., Lüders S., Murray G.D., Richter P.S., Roine R.O., Terént A. (2011). The angiotensin-receptor blocker candesartan for treatment of acute stroke (SCAST): A randomised, placebo-controlled, double-blind trial. Lancet.

[27] ENOS Trial Investigators (2015). Efficacy of nitric oxide, with or without continuing antihypertensive treatment, for management of high blood pressure in acute stroke (ENOS): A partial-factorial randomised controlled trial. Lancet.

[28] He J., Zhang Y., Xu T., Zhao Q., Wang D., Chen C.S., Tong W., Liu C., Xu T., Ju Z. (2014). Effects of immediate blood pressure reduction on death and major disability in patients with acute ischemic stroke: The CATIS randomized clinical trial. JAMA.

[29] Bath P.M., Martin R.H., Palesch Y., Cotton D., Yusuf S., Sacco R., Diener H.C., Toni D., Estol C., Roberts R. (2009). Effect of telmisartan on functional outcome, recurrence, and blood pressure in patients with acute mild ischemic stroke: A PRoFESS subgroup analysis. Stroke.

[30] Wahlgren N.G., MacMahon D.G., De Keyser J., Indredavik B., Ryman T. (1994). Intravenous Nimodipine West European Stroke Trial (INWEST) of Nimodipine in the Treatment of Acute Ischaemic Stroke. Cerebrovasc. Dis..

[31] Bang O.Y., Chung J.W., Kim S.K., Kim S.J., Lee M.J., Hwang J., Seo W.K., Ha Y.S., Sung S.M., Kim E.G. (2019). Therapeutic-induced hypertension in patients with noncardioembolic acute stroke. Neurology.

[32] Kang J., Hong J.H., Jang M.U., Choi N.C., Lee J.S., Kim B.J., Han M.K., Bae H.J. (2017). Change in blood pressure variability in patients with acute ischemic stroke and its effect on early neurologic outcome. PLoS ONE.

[33] Chung J.W., Kim N., Kang J., Park S.H., Kim W.J., Ko Y., Park J.H., Lee J.S., Lee J., Yang M.H. (2015). Blood pressure variability and the development of early neurological deterioration following acute ischemic stroke. J. Hypertens..

[34] Stead L.G., Gilmore R.M., Vedula K.C., Weaver A.L., Decker W.W., Brown R.D. (2006). Impact of acute blood pressure variability on ischemic stroke outcome. Neurology.

[35] de Havenon A., Stoddard G., Saini M., Wong K.H., Tirschwell D., Bath P. (2019). Increased blood pressure variability after acute ischemic stroke increases the risk of death: A secondary analysis of the Virtual International Stroke Trial Archive. JRSM Cardiovasc. Dis..

[36] Woods A.G., Lillicrap T., Hood R., Fletcher J.W., Ranhage V., Larsson E., Cahlin F., Jood K., Tatlisumak T., Garcia-Esperon C. (2023). Blood Pressure Variability Is Associated with Infarct Growth in Acute Ischaemic Stroke. Cerebrovasc. Dis..

[37] Wu M.N., Liu Y.P., Fong Y.O., Lin Y.H., Yang I.H., Chou P.S., Hsu C.Y., Lin H.F. (2024). The impact of blood pressure variability on the development of parenchymal hematoma in acute cerebral infarction with atrial fibrillation. Hypertens. Res..

[38] Chen Y., Ma Y., Qin J., Wei X., Yang Y., Yuan Y., Yan F., Huo X., Han L. (2023). Blood pressure variability predicts poor outcomes in acute stroke patients without thrombolysis: A systematic review and meta-analysis. J. Neurol..

[39] Todo K. (2024). Blood pressure variability in acute ischemic stroke. Hypertens. Res..

[40] Polychronopoulos G., Milonas D., Tziomalos K. (2023). Blood Pressure Variability in Patients with Acute Ischemic Stroke: Is It Worth Measuring?. Am. J. Hypertens..

[41] Yuan F., Yang F., Zhao J., Fu F., Liu Y., Xue C., Wang K., Yuan X., Li D., Liu Q. (2021). Controlling Hypertension after Severe Cerebrovascular Event (CHASE): A randomized, multicenter, controlled study. Int. J. Stroke.

[42] Zhao J., Yuan F., Fu F., Liu Y., Xue C., Wang K., Yuan X., Li D., Liu Q., Zhang W. (2021). Blood pressure variability and outcome in acute severe stroke: A post hoc analysis of CHASE-A randomized controlled trial. J. Clin. Hypertens..

[43] Fukuda K., Kai H., Kamouchi M., Hata J., Ago T., Nakane H., Imaizumi T., Kitazono T. (2015). Day-by-Day Blood Pressure Variability and Functional Outcome after Acute Ischemic Stroke: Fukuoka Stroke Registry. Stroke.

[44] Manning L.S., Mistri A.K., Potter J., Rothwell P.M., Robinson T.G. (2015). Short-term blood pressure variability in acute stroke: Post hoc analysis of the controlling hypertension and hypotension immediately post stroke and continue or stop post-stroke antihypertensives collaborative study trials. Stroke.

[45] Minhas J.S., Wang X., Lavados P.M., Moullaali T.J., Arima H., Billot L., Hackett M.L., Olavarria V.V., Middleton S., Pontes-Neto O. (2019). Blood pressure variability and outcome in acute ischemic and hemorrhagic stroke: A post hoc analysis of the HeadPoST study. J. Hum. Hypertens..

[46] Shin D.H., Song S., Lee Y.B. (2019). Comparison of the Effect of Fimasartan versus Valsartan on Blood Pressure Variability in Acute Ischemic Stroke: A Double-Blind Randomized Trial. Cardiovasc. Ther..

[47] Robinson T.G., Davison W.J., Rothwell P.M., Potter J.F. (2019). Randomised controlled trial of a Calcium Channel or Angiotensin Converting Enzyme Inhibitor/Angiotensin Receptor Blocker Regime to Reduce Blood Pressure Variability following Ischaemic Stroke (CAARBS): A protocol for a feasibility study. BMJ Open.

[48] Davison W.J., Appiah K., Robinson T.G., McGurgan I.J., Rothwell P.M., Potter J.F. (2020). A calcium channel or angiotensin converting enzyme inhibitor/angiotensin receptor blocker regime to reduced blood pressure variability in acute ischaemic stroke (CAARBS): A feasibility trial. J. Neurol. Sci..

[49] Tsivgoulis G., Saqqur M., Sharma V.K., Lao A.Y., Hill M.D., Alexandrov A.V. (2007). Association of pretreatment blood pressure with tissue plasminogen activator-induced arterial recanalization in acute ischemic stroke. Stroke.

[50] Martin-Schild S., Hallevi H., Albright K.C., Khaja A.M., Barreto A.D., Gonzales N.R., Grotta J.C., Savitz S.I. (2008). Aggressive blood pressure-lowering treatment before intravenous tissue plasminogen activator therapy in acute ischemic stroke. Arch. Neurol..

[51] Sandset E.C. (2014). Blood pressure in acute stroke. Lancet Neurol..

[52] Malhotra K., Ahmed N., Filippatou A., Katsanos A.H., Goyal N., Tsioufis K., Manios E., Pikilidou M., Schellinger P.D., Alexandrov A.W. (2019). Association of Elevated Blood Pressure Levels with Outcomes in Acute Ischemic Stroke Patients Treated with Intravenous Thrombolysis: A Systematic Review and Meta-Analysis. J. Stroke.

[53] Tsivgoulis G., Kotsis V., Giannopoulos S. (2011). Intravenous thrombolysis for acute ischaemic stroke: Effective blood pressure control matters. Int. J. Stroke.

[54] Anderson C.S., Huang Y., Lindley R.I., Chen X., Arima H., Chen G., Li Q., Billot L., Delcourt C., Bath P.M. (2019). Intensive blood pressure reduction with intravenous thrombolysis therapy for acute ischaemic stroke (ENCHANTED): An international, randomised, open-label, blinded-endpoint, phase 3 trial. Lancet.

[55] Sandset E.C., Fischer U. (2019). Blood pressure lowering in acute ischaemic stroke thrombolysis. Lancet.

[56] Thomalla G., Simonsen C.Z., Boutitie F., Andersen G., Berthezene Y., Cheng B., Cheripelli B., Cho T.H., Fazekas F., Fiehler J. (2018). MRI-Guided Thrombolysis for Stroke with Unknown Time of Onset. N. Engl. J. Med..

[57] Barow E., Boutitie F., Cheng B., Cho T.H., Ebinger M., Endres M., Fiebach J.B., Fiehler J., Nickel A., Puig J. (2021). 24-hour blood pressure variability and treatment effect of intravenous alteplase in acute ischaemic stroke. Eur. Stroke J..

[58] Kellert L., Sykora M., Gumbinger C., Herrmann O., Ringleb P.A. (2012). Blood pressure variability after intravenous thrombolysis in acute stroke does not predict intracerebral hemorrhage but poor outcome. Cerebrovasc. Dis..

[59] Kellert L., Hametner C., Ahmed N., Rauch G., MacLeod M.J., Perini F., Lees K.R., Ringleb P.A. (2017). Reciprocal Interaction of 24-Hour Blood Pressure Variability and Systolic Blood Pressure on Outcome in Stroke Thrombolysis. Stroke.

[60] Delgado-Mederos R., Ribo M., Rovira A., Rubiera M., Munuera J., Santamarina E., Delgado P., Maisterra O., Alvarez-Sabin J., Molina C.A. (2008). Prognostic significance of blood pressure variability after thrombolysis in acute stroke. Neurology.

[61] Endo K., Kario K., Koga M., Nakagawara J., Shiokawa Y., Yamagami H., Furui E., Kimura K., Hasegawa Y., Okada Y. (2013). Impact of early blood pressure variability on stroke outcomes after thrombolysis: The SAMURAI rt-PA Registry. Stroke.

[62] Reddy S., Paramasivan N.K., Sreedharan S.E., Sukumaran S., Vinoda Thulaseedharan J., Sylaja P.N. (2023). Association of 24 h Blood Pressure on Functional Outcome in Patients with Acute Ischemic Stroke Post Intravenous Thrombolysis. Cerebrovasc. Dis..

[63] Wang Z.X., Wang C., Zhang P., Qu Y., Guo Z.N., Yang Y. (2020). Effects of Early Changes in Blood Pressure During Intravenous Thrombolysis on the Prognosis of Acute Ischemic Stroke Patients. Front. Aging Neurosci..

[64] Liu K., Yan S., Zhang S., Guo Y., Lou M. (2016). Systolic Blood Pressure Variability is Associated with Severe Hemorrhagic Transformation in the Early Stage After Thrombolysis. Transl. Stroke Res..

[65] He M., Wang H., Tang Y., Wang J., Cui B., Xu B., Sun Y., Zhang G., He X., Niu X. (2022). Blood pressure undulation of peripheral thrombolysis period in acute ischemic stroke is associated with prognosis. J. Hypertens..

[66] Katsanos A.H., Alexandrov A.V., Mandava P., Köhrmann M., Soinne L., Barreto A.D., Sharma V.K., Mikulik R., Muir K.W., Rothlisberger T. (2020). Pulse pressure variability is associated with unfavorable outcomes in acute ischaemic stroke patients treated with intravenous thrombolysis. Eur. J. Neurol..

[67] Wei X., Duan Z., Zhai Y., Zhang C., Zhang J., Hu T., Liu T., Liu Z., Xu J., Liu H. (2022). Early blood pressure changes during systemic thrombolysis and its association with unexplained early neurological deterioration in small subcortical infarct. J. Clin. Hypertens..

[68] Yao M.X., Qiu D.H., Zheng W.C., Zhao J.H., Yin H.P., Liu Y.L., Chen Y.K. (2022). Effects of Early-Stage Blood Pressure Variability on the Functional Outcome in Acute Ischemic Stroke Patients With Symptomatic Intracranial Artery Stenosis or Occlusion Receiving Intravenous Thrombolysis. Front. Neurol..

[69] Malhotra K., Goyal N., Katsanos A.H., Filippatou A., Mistry E.A., Khatri P., Anadani M., Spiotta A.M., Sandset E.C., Sarraj A. (2020). Association of Blood Pressure with Outcomes in Acute Stroke Thrombectomy. Hypertension.

[70] Goyal N., Tsivgoulis G., Iftikhar S., Khorchid Y., Fawad Ishfaq M., Doss V.T., Zand R., Chang J., Alsherbini K., Choudhri A. (2017). Admission systolic blood pressure and outcomes in large vessel occlusion strokes treated with endovascular treatment. J. Neurointerventional. Surg..

[71] Mulder M., Ergezen S., Lingsma H.F., Berkhemer O.A., Fransen P.S.S., Beumer D., van den Berg L.A., Lycklama À Nijeholt G., Emmer B.J., van der Worp H.B. (2017). Baseline Blood Pressure Effect on the Benefit and Safety of Intra-Arterial Treatment in MR CLEAN (Multicenter Randomized Clinical Trial of Endovascular Treatment of Acute Ischemic Stroke in The Netherlands). Stroke.

[72] Maïer B., Gory B., Taylor G., Labreuche J., Blanc R., Obadia M., Abrivard M., Smajda S., Desilles J.P., Redjem H. (2017). Mortality and Disability According to Baseline Blood Pressure in Acute Ischemic Stroke Patients Treated by Thrombectomy: A Collaborative Pooled Analysis. J. Am. Heart Assoc..

[73] Petersen N.H., Ortega-Gutierrez S., Wang A., Lopez G.V., Strander S., Kodali S., Silverman A., Zheng-Lin B., Dandapat S., Sansing L.H. (2019). Decreases in Blood Pressure During Thrombectomy Are Associated with Larger Infarct Volumes and Worse Functional Outcome. Stroke.

[74] Löwhagen Hendén P., Rentzos A., Karlsson J.E., Rosengren L., Sundeman H., Reinsfelt B., Ricksten S.E. (2015). Hypotension during Endovascular Treatment of Ischemic Stroke Is a Risk Factor for Poor Neurological Outcome. Stroke.

[75] Whalin M.K., Halenda K.M., Haussen D.C., Rebello L.C., Frankel M.R., Gershon R.Y., Nogueira R.G. (2017). Even Small Decreases in Blood Pressure during Conscious Sedation Affect Clinical Outcome after Stroke Thrombectomy: An Analysis of Hemodynamic Thresholds. AJNR Am. J. Neuroradiol..

[76] Maïer B., Fahed R., Khoury N., Guenego A., Labreuche J., Taylor G., Blacher J., Zuber M., Lapergue B., Blanc R. (2019). Association of Blood Pressure during Thrombectomy for Acute Ischemic Stroke with Functional Outcome: A Systematic Review. Stroke.

[77] Rasmussen M., Schönenberger S., Hendèn P.L., Valentin J.B., Espelund U.S., Sørensen L.H., Juul N., Uhlmann L., Johnsen S.P., Rentzos A. (2020). Blood Pressure Thresholds and Neurologic Outcomes after Endovascular Therapy for Acute Ischemic Stroke: An Analysis of Individual Patient Data from 3 Randomized Clinical Trials. JAMA Neurol..

[78] Martins A.I., Sargento-Freitas J., Silva F., Jesus-Ribeiro J., Correia I., Gomes J.P., Aguiar-Gonçalves M., Cardoso L., Machado C., Rodrigues B. (2016). Recanalization Modulates Association Between Blood Pressure and Functional Outcome in Acute Ischemic Stroke. Stroke.

[79] Mazighi M., Richard S., Lapergue B., Sibon I., Gory B., Berge J., Consoli A., Labreuche J., Olivot J.M., Broderick J. (2021). Safety and efficacy of intensive blood pressure lowering after successful endovascular therapy in acute ischaemic stroke (BP-TARGET): A multicentre, open-label, randomised controlled trial. Lancet Neurol..

[80] Yang P., Song L., Zhang Y., Zhang X., Chen X., Li Y., Sun L., Wan Y., Billot L., Li Q. (2022). Intensive blood pressure control after endovascular thrombectomy for acute ischaemic stroke (ENCHANTED2/MT): A multicentre, open-label, blinded-endpoint, randomised controlled trial. Lancet.

[81] Nam H.S., Kim Y.D., Heo J., Lee H., Jung J.W., Choi J.K., Lee I.H., Lim I.H., Hong S.H., Baik M. (2023). Intensive vs Conventional Blood Pressure Lowering After Endovascular Thrombectomy in Acute Ischemic Stroke: The OPTIMAL-BP Randomized Clinical Trial. JAMA.

[82] Mistry E.A., Hart K.W., Davis L.T., Gao Y., Prestigiacomo C.J., Mittal S., Mehta T., LaFever H., Harker P., Wilson-Perez H.E. (2023). Blood Pressure Management After Endovascular Therapy for Acute Ischemic Stroke: The BEST-II Randomized Clinical Trial. JAMA.

[83] Chen M., Meis J., Potreck A., Sauer L.D., Kieser M., Bendszus M., Wick W., Ringleb P.A., Möhlenbruch M.A., Schönenberger S. (2023). Effect of Individualized Versus Standardized Blood Pressure Management During Endovascular Stroke Treatment on Clinical Outcome: A Randomized Clinical Trial. Stroke.

[84] Katsanos A.H., Catanese L., Sahlas D.J., Srivastava A., Veroniki A.A., Perera K., Ng K.K.H., Joundi R., Adel B.V., Larrazabal R. (2024). Blood Pressure Management Following Endovascular Stroke Treatment: A Feasibility Trial and Meta-Analysis of Outcomes. Stroke Vasc. Interv. Neurol..

[85] Maïer B., Gory B., Lapergue B., Sibon I., Escalard S., Kyheng M., Labreuche J., de Havenon A., Petersen N., Anadani M. (2022). Effect of blood pressure variability in the randomized controlled BP TARGET trial. Eur. J. Neurol..

[86] Palaiodimou L., Joundi R.A., Katsanos A.H., Ahmed N., Kim J.T., Goyal N., Maier I.L., de Havenon A., Anadani M., Matusevicius M. (2023). Association between blood pressure variability and outcomes after endovascular thrombectomy for acute ischemic stroke: An individual patient data meta-analysis. Eur. Stroke J..

[87] Yang M., Lu T., Weng B., He Y., Yang H. (2021). Association Between Blood Pressure Variability and Short-Term Outcome after Intra-arterial Thrombectomy in Acute Stroke Patients with Large-Vessel Occlusion. Front. Neurol..

[88] Mistry E.A., Mehta T., Mistry A., Arora N., Starosciak A.K., De Los Rios La Rosa F., Siegler J.E., Chitale R., Anadani M., Yaghi S. (2020). Blood Pressure Variability and Neurologic Outcome after Endovascular Thrombectomy: A Secondary Analysis of the BEST Study. Stroke.

[89] Qin J., He Q., Zhang Z. (2022). Detrimental effect of increased blood pressure variability on clinical outcome in acute ischemic stroke treated with reperfusion therapy: A case control study. BMC Neurol..

[90] Zhang T., Wang X., Wen C., Zhou F., Gao S., Zhang X., Lin S., Shi J., Li W. (2019). Effect of short-term blood pressure variability on functional outcome after intra-arterial treatment in acute stroke patients with large-vessel occlusion. BMC Neurol..

[91] Xu C., Jin T., Chen Z., Zhang Z., Zhang K., Mao H., Ye S., Geng Y., Shi Z. (2022). Increased blood pressure variability during general anaesthesia is associated with worse outcomes after mechanical thrombectomy: A prospective observational cohort study. BMJ Open.

[92] Lu Y., Shen R., Lin W., Zhou X., Hu J., Zhang Q. (2022). Association between blood pressure variability and clinical outcomes after successful recanalization in patients with large vessel occlusion stroke after mechanical thrombectomy. Front. Neurol..

[93] Nepal G., Shrestha G.S., Shing Y.K., Muha A., Bhagat R. (2021). Systolic blood pressure variability following endovascular thrombectomy and clinical outcome in acute ischemic stroke: A meta-analysis. Acta Neurol. Scand..

[94] Goyal N., Tsivgoulis G., Pandhi A., Chang J.J., Dillard K., Ishfaq M.F., Nearing K., Choudhri A.F., Hoit D., Alexandrov A.W. (2017). Blood pressure levels post mechanical thrombectomy and outcomes in large vessel occlusion strokes. Neurology.

[95] Maier I.L., Tsogkas I., Behme D., Bähr M., Knauth M., Psychogios M.N., Liman J. (2018). High Systolic Blood Pressure after Successful Endovascular Treatment Affects Early Functional Outcome in Acute Ischemic Stroke. Cerebrovasc. Dis..

[96] Matusevicius M., Cooray C., Bottai M., Mazya M., Tsivgoulis G., Nunes A.P., Moreira T., Ollikainen J., Tassi R., Strbian D. (2020). Blood Pressure After Endovascular Thrombectomy: Modeling for Outcomes Based on Recanalization Status. Stroke.

[97] Cho B.H., Kim J.T., Lee J.S., Park M.S., Kang K.W., Choi K.H., Lee S.H., Choi S.M., Kim B.C., Kim M.K. (2019). Associations of various blood pressure parameters with functional outcomes after endovascular thrombectomy in acute ischaemic stroke. Eur. J. Neurol..

[98] Bennett A.E., Wilder M.J., McNally J.S., Wold J.J., Stoddard G.J., Majersik J.J., Ansari S., de Havenon A. (2018). Increased blood pressure variability after endovascular thrombectomy for acute stroke is associated with worse clinical outcome. J. Neurointerventional Surg..

[99] Kim T.J., Park H.K., Kim J.M., Lee J.S., Park S.H., Jeong H.B., Park K.Y., Rha J.H., Yoon B.W., Ko S.B. (2019). Blood pressure variability and hemorrhagic transformation in patients with successful recanalization after endovascular recanalization therapy: A retrospective observational study. Ann. Neurol..

[100] Anadani M., Orabi Y., Alawieh A., Chatterjee A., Lena J., Al Kasab S., Spiotta A.M. (2019). Blood pressure and outcome post mechanical thrombectomy. J. Clin. Neurosci..

[101] Chu H.J., Lin C.H., Chen C.H., Hwang Y.T., Lee M., Lee C.W., Tang S.C., Jeng J.S. (2020). Effect of blood pressure parameters on functional independence in patients with acute ischemic stroke in the first 6 hours after endovascular thrombectomy. J. Neurointerventional Surg..

[102] Zhou J., Samara H., Ebrahim A., Kinariwala J., Mohamed W. (2023). Blood pressure variability and short-term outcomes after mechanical thrombectomy in patients with acute ischemic stroke. J. Stroke Cerebrovasc. Dis..

[103] Prasad A., Kobsa J., Kodali S., Bartolome D., Begunova L., Quispe-Orozco D., Farooqui M., Zevallos C., Ortega-Gutiérrez S., Anadani M. (2022). Temporal profiles of systolic blood pressure variability and neurologic outcomes after endovascular thrombectomy. Eur. Stroke J..

[104] Anadani M., Orabi M.Y., Alawieh A., Goyal N., Alexandrov A.V., Petersen N., Kodali S., Maier I.L., Psychogios M.N., Swisher C.B. (2019). Blood Pressure and Outcome after Mechanical Thrombectomy with Successful Revascularization. Stroke.

[105] Mujanovic A., Ng F., Meinel T.R., Dobrocky T., Piechowiak E.I., Kurmann C.C., Seiffge D.J., Wegener S., Wiest R., Meyer L. (2024). No-reflow phenomenon in stroke patients: A systematic literature review and meta-analysis of clinical data. Int. J. Stroke.

[106] Nie X., Leng X., Miao Z., Fisher M., Liu L. (2023). Clinically Ineffective Reperfusion After Endovascular Therapy in Acute Ischemic Stroke. Stroke.

[107] Jillella D.V., Calder C.S., Uchino K., Qeadan F., Ikram A., Casul Y.R., Tran H.Q. (2020). Blood Pressure and Hospital Discharge Outcomes in Acute Ischemic Stroke Patients Undergoing Reperfusion Therapy. J. Stroke Cerebrovasc. Dis..

[108] Koshimizu H., Kojima R., Kario K., Okuno Y. (2020). Prediction of blood pressure variability using deep neural networks. Int. J. Med. Inform..

[109] Najafali D., Johnstone T., Pergakis M., Buganu A., Ullah M., Vuong K., Panchal B., Sutherland M., Yarbrough K.L., Phipps M.S. (2023). Prediction of blood pressure variability during thrombectomy using supervised machine learning and outcomes of patients with ischemic stroke from large vessel occlusion. J. Thromb. Thrombolysis.

[110] Castro P., Ferreira F., Nguyen C.K., Payabvash S., Ozan Tan C., Sorond F., Azevedo E., Petersen N. (2021). Rapid Assessment of Blood Pressure Variability and Outcome After Successful Thrombectomy. Stroke.

[111] Jiang X., Gao L., Wang J., Bao J., Fang J., He L. (2023). Collateral Status Modification of the Association Between Blood Pressure Variation Within 72 Hours After Endovascular Treatment and Clinical Outcome in Acute Ischemic Stroke: A Retrospective Cohort Study. Clin. Interv. Aging.

[112] Liu D., Nie X., Pan Y., Yan H., Pu Y., Wei Y., Cai Y., Ding Y., Lu Q., Zhang Z. (2021). Adverse Outcomes Associated With Higher Mean Blood Pressure and Greater Blood Pressure Variability Immediately After Successful Embolectomy in Those With Acute Ischemic Stroke, and the Influence of Pretreatment Collateral Circulation Status. J. Am. Heart Assoc..

[113] Chang J.Y., Jeon S.B., Jung C., Gwak D.S., Han M.K. (2019). Postreperfusion Blood Pressure Variability after Endovascular Thrombectomy Affects Outcomes in Acute Ischemic Stroke Patients with Poor Collateral Circulation. Front. Neurol..

[114] Huang X., Guo H., Yuan L., Cai Q., Zhang M., Zhang Y., Zhu W., Li Z., Yang Q., Zhou Z. (2021). Blood pressure variability and outcomes after mechanical thrombectomy based on the recanalization and collateral status. Ther. Adv. Neurol. Disord..

[115] Chang J.Y., Jeon S.B., Lee J.H., Kwon O.K., Han M.K. (2018). The Relationship between Blood Pressure Variability, Recanalization Degree, and Clinical Outcome in Large Vessel Occlusive Stroke after an Intra-Arterial Thrombectomy. Cerebrovasc. Dis..

[116] Petersen N.H., Silverman A., Strander S.M., Kodali S., Wang A., Sansing L.H., Schindler J.L., Falcone G.J., Gilmore E.J., Jasne A.S. (2020). Fixed Compared With Autoregulation-Oriented Blood Pressure Thresholds After Mechanical Thrombectomy for Ischemic Stroke. Stroke.

[117] Tao C., Xu P., Yao Y., Zhu Y., Li R., Li J., Luo W., Hu W. (2021). A Prospective Study to Investigate Controlling Blood Pressure Under Transcranial Doppler after Endovascular Treatment in Patients with Occlusion of Anterior Circulation. Front. Neurol..

[118] Webb A.J., Rothwell P.M. (2011). Effect of dose and combination of antihypertensives on interindividual blood pressure variability: A systematic review. Stroke.

[119] Webb A.J., Fischer U., Mehta Z., Rothwell P.M. (2010). Effects of antihypertensive-drug class on interindividual variation in blood pressure and risk of stroke: A systematic review and meta-analysis. Lancet.

[120] Li J., Liu B.J., Wang Y.J., Cui Y., Chen H.S. (2024). Antihypertensive Drugs Affect the Association of Systolic Blood Pressure Variability with Outcomes in Patients with Acute Stroke who had Successful Recanalization after Endovascular Treatment. J. Atheroscler. Thromb..

[121] Parati G., Ravogli A., Frattola A., Groppelli A., Ulian L., Santucciu C., Mancia G. (1994). Blood pressure variability: Clinical implications and effects of antihypertensive treatment. J. Hypertens. Suppl..

[122] Liu H.T., Deng N.H., Wu Z.F., Zhou Z.Y., Tian Z., Liu X.Y., Wang Y.X., Zheng H.Y., Ou Y.S., Jiang Z.S. (2023). Statin’s role on blood pressure levels: Meta-analysis based on randomized controlled trials. J. Clin. Hypertens..

[123] Cordonnier C., Demchuk A., Ziai W., Anderson C.S. (2018). Intracerebral haemorrhage: Current approaches to acute management. Lancet.

[124] Ma L., Hu X., Song L., Chen X., Ouyang M., Billot L., Li Q., Malavera A., Li X., Muñoz-Venturelli P. (2023). The third Intensive Care Bundle with Blood Pressure Reduction in Acute Cerebral Haemorrhage Trial (INTERACT3): An international, stepped wedge cluster randomised controlled trial. Lancet.

[125] Anderson C.S., Heeley E., Huang Y., Wang J., Stapf C., Delcourt C., Lindley R., Robinson T., Lavados P., Neal B. (2013). Rapid blood-pressure lowering in patients with acute intracerebral hemorrhage. N. Engl. J. Med..

[126] Manning L., Hirakawa Y., Arima H., Wang X., Chalmers J., Wang J., Lindley R., Heeley E., Delcourt C., Neal B. (2014). Blood pressure variability and outcome after acute intracerebral haemorrhage: A post-hoc analysis of INTERACT2, a randomised controlled trial. Lancet Neurol..

[127] Divani A.A., Liu X., Di Napoli M., Lattanzi S., Ziai W., James M.L., Jafarli A., Jafari M., Saver J.L., Hemphill J.C. (2019). Blood Pressure Variability Predicts Poor In-Hospital Outcome in Spontaneous Intracerebral Hemorrhage. Stroke.

[128] Tanaka E., Koga M., Kobayashi J., Kario K., Kamiyama K., Furui E., Shiokawa Y., Hasegawa Y., Okuda S., Todo K. (2014). Blood pressure variability on antihypertensive therapy in acute intracerebral hemorrhage: The Stroke Acute Management with Urgent Risk-factor Assessment and Improvement-intracerebral hemorrhage study. Stroke.

[129] Meeks J.R., Bambhroliya A.B., Meyer E.G., Slaughter K.B., Fraher C.J., Sharrief A.Z., Bowry R., Ahmed W.O., Tyson J.E., Miller C.C. (2019). High in-hospital blood pressure variability and severe disability or death in primary intracerebral hemorrhage patients. Int. J. Stroke.

[130] Rodriguez-Luna D., Piñeiro S., Rubiera M., Ribo M., Coscojuela P., Pagola J., Flores A., Muchada M., Ibarra B., Meler P. (2013). Impact of blood pressure changes and course on hematoma growth in acute intracerebral hemorrhage. Eur. J. Neurol..

[131] Lattanzi S., Cagnetti C., Provinciali L., Silvestrini M. (2015). Blood Pressure Variability and Clinical Outcome in Patients with Acute Intracerebral Hemorrhage. J. Stroke Cerebrovasc. Dis..

[132] de Havenon A., Majersik J.J., Stoddard G., Wong K.H., McNally J.S., Smith A.G., Rost N.S., Tirschwell D.L. (2018). Increased Blood Pressure Variability Contributes to Worse Outcome After Intracerebral Hemorrhage. Stroke.

[133] Qu Y., Guo Z.N., Zhang P., Ma H.Y., Sun Y.Y., Ren J.X., Liu J., Zhang P.D., Yang Y. (2022). Time course of beat-to-beat blood pressure variability and outcome in patients with spontaneous intracerebral haemorrhage. J. Hypertens..

[134] Zhang H.X., Fan Q.X., Xue S.Z., Zhang M., Zhao J.X. (2018). Twenty-four-hour blood pressure variability plays a detrimental role in the neurological outcome of hemorrhagic stroke. J. Int. Med. Res..

[135] Jeon J.P., Kim C., Kim S.E. (2018). Blood Pressure Variability and Outcome in Patients with Acute Nonlobar Intracerebral Hemorrhage following Intensive Antihypertensive Treatment. Chin. Med. J..

[136] Liu W., Zhuang X., Zhang L. (2021). Prognostic Value of Blood Pressure Variability for Patients With Acute or Subacute Intracerebral Hemorrhage: A Meta-Analysis of Prospective Studies. Front. Neurol..

[137] Moullaali T.J., Wang X., Martin R.H., Shipes V.B., Robinson T.G., Chalmers J., Suarez J.I., Qureshi A.I., Palesch Y.Y., Anderson C.S. (2019). Blood pressure control and clinical outcomes in acute intracerebral haemorrhage: A preplanned pooled analysis of individual participant data. Lancet Neurol..

[138] Andalib S., Lattanzi S., Di Napoli M., Petersen A., Biller J., Kulik T., Macri E., Girotra T., Torbey M.T., Divani A.A. (2020). Blood Pressure Variability: A New Predicting Factor for Clinical Outcomes of Intracerebral Hemorrhage. J. Stroke Cerebrovasc. Dis..

[139] Qureshi A.I., Qureshi M.H. (2018). Acute hypertensive response in patients with intracerebral hemorrhage pathophysiology and treatment. J. Cereb. Blood Flow. Metab..

[140] Vemmos K.N., Tsivgoulis G., Spengos K., Zakopoulos N., Synetos A., Kotsis V., Vassilopoulos D., Mavrikakis M. (2003). Association between 24-h blood pressure monitoring variables and brain oedema in patients with hyperacute stroke. J. Hypertens..

[141] Sykora M., Diedler J., Turcani P., Rupp A., Steiner T. (2009). Subacute perihematomal edema in intracerebral hemorrhage is associated with impaired blood pressure regulation. J. Neurol. Sci..

[142] Olivot J.M., Mlynash M., Kleinman J.T., Straka M., Venkatasubramanian C., Bammer R., Moseley M.E., Albers G.W., Wijman C.A. (2010). MRI profile of the perihematomal region in acute intracerebral hemorrhage. Stroke.

[143] Menon R.S., Burgess R.E., Wing J.J., Gibbons M.C., Shara N.M., Fernandez S., Jayam-Trouth A., German L., Sobotka I., Edwards D. (2012). Predictors of highly prevalent brain ischemia in intracerebral hemorrhage. Ann. Neurol..

[144] Kang K., Shi K., Liu J., Li N., Wu J., Zhao X. (2024). Autonomic dysfunction and treatment strategies in intracerebral hemorrhage. CNS Neurosci. Ther..

[145] Jafari M., Damani R. (2020). Blood pressure variability and outcome after acute intracerebral hemorrhage. J. Neurol. Sci..

[146] Rabinstein A.A., Anderson C.D. (2015). Time is brain also counts for ICH. Neurology.

[147] Graffagnino C., Bergese S., Love J., Schneider D., Lazaridis C., LaPointe M., Lee K., Lynch G., Hu M.Y., Williams G.C. (2013). Clevidipine rapidly and safely reduces blood pressure in acute intracerebral hemorrhage: The ACCELERATE trial. Cerebrovasc. Dis..

[148] Poyant J.O., Kuper P.J., Mara K.C., Dierkhising R.A., Rabinstein A.A., Wijdicks E.F.M., Ritchie B.M. (2019). Nicardipine Reduces Blood Pressure Variability After Spontaneous Intracerebral Hemorrhage. Neurocrit. Care.

[149] Lin Q.S., Ping C., Lin Y.X., Lin Z.Y., Yu L.H., Dai L.S., Kang D.Z. (2016). Systolic Blood Pressure Variability is a Novel Risk Factor for Rebleeding in Acute Subarachnoid Hemorrhage: A Case-Control Study. Medicine.

[150] Yang M., Pan X., Liang Z., Huang X., Duan M., Cai H., Jiang G., Wen X., Chen L. (2019). Association between blood pressure variability and the short-term outcome in patients with acute spontaneous subarachnoid hemorrhage. Hypertens. Res..

[151] Beseoglu K., Unfrau K., Steiger H.J., Hänggi D. (2010). Influence of blood pressure variability on short-term outcome in patients with subarachnoid hemorrhage. Cent. Eur. Neurosurg..

[152] Kirkness C.J., Burr R.L., Mitchell P.H. (2009). Intracranial and blood pressure variability and long-term outcome after aneurysmal sub-arachnoid hemorrhage. Am. J. Crit. Care.

[153] Igase M., Igase K., Kohara K., Yamashita S., Fujisawa M., Katagi R., Miki T. (2013). Visit-to-visit variability in systolic blood pressure is a novel risk factor for the growth of intracranial aneurysms. Cerebrovasc. Dis..

